# Positive regulators of T cell proliferation as biomarkers for predicting prognosis and characterizing the immune landscape in lung adenocarcinoma

**DOI:** 10.3389/fgene.2022.1003754

**Published:** 2022-11-25

**Authors:** Yang Li, Gang Peng, Chaoying Qin, Xiangyu Wang, Yue Li, Yueran Li

**Affiliations:** ^1^ Department of Laboratory Medicine, Third Xiangya Hospital, Central South University, Changsha, Hunan, China; ^2^ Department of Neurosurgery, Xiangya Hospital, Central South University, Changsha, Hunan, China; ^3^ Department of Gynecology, Third Xiangya Hospital, Central South University, Changsha, Hunan, China

**Keywords:** lung adenocarcinoma, driver of T cell proliferation, tumor microenvironment, prognosis, immune prognostic model

## Abstract

Lung adenocarcinoma (LUAD) is the one of the most prevalent and fatal form of malignant tumors worldwide. Recently, immunotherapy is widely used in the treatment of patients with LUAD and has proved to be clinically effective in improve the prognosis of patients. But there still has been a tremendous thrust to further improve the efficacy of immunotherapy in individual patients with LUAD. The suppression of T cells and their effector functions in the tumor microenvironment (TME) of LUAD is one of the primary reasons for the low efficacy of immunotherapy in some patients with LUAD. Therefore, identifying positive regulators of T cell proliferation (TPRs) may offer novel avenues for LUAD immunotherapy. In this study, we comprehensively evaluated the infiltration patterns of TPRs in 1,066 patients with LUAD using unsupervised consensus clustering and identified correlations with genomic and clinicopathological characteristics. Three infiltrating TPR clusters were defined, and a TPR-related risk signature composed of nine TPRs was constructed using least absolute shrinkage and selection operator-Cox regression algorithms to classify the individual TPR infiltration patterns. Cluster 1 exhibited high levels of T cell infiltration and activation of immune-related signaling pathways, whereas cluster 2 was characterized by robust T cell immune infiltration and enrichment of pathways associated with carcinogenic gene sets and tumor immunity. Cluster 3 was characterized as an immune-desert phenotype. Moreover, the TPR signature was confirmed as an independent prognostic biomarker for drug sensitivity in patients with LUAD. In conclusion, the TPR signature may serve as a novel tool for effectively characterizing immune characteristics and evaluating the prognosis of patients with LUAD.

## Introduction

Lung cancer is the most frequently diagnosed and most lethal cancer worldwide, with a 5-year relative survival rate of 21% (Siegel et al., 2021). Lung adenocarcinoma (LUAD) is the most common pathological type of non-small cell lung cancer (NSCLC), accounting for 50% of lung cancers (Travis, 2011; Brustugun et al., 2018). Surgery remains the primary treatment for patients with stage I LUAD, but the prognosis remains poor, owing to the prevalence of metastasis before diagnosis (Herbst et al., 2018). The risk of recurrence 5 years after surgery is as high as 27% (Yan et al., 2009). Recent advances in targeted therapies for driver genes of LUAD may reduce metastasis, delay postoperative recurrence, and improve patient survival rates (Mayekar and Bivona, 2017). For example, targeted therapies that employ epidermal growth factor receptor (EGFR) tyrosine kinase inhibitors (TKIs) against tumors with EGFR mutations or anaplastic lymphoma kinase (ALK) TKIs against tumors with ALK fusions have improved the outcomes in a subset of patients (Saito et al., 2018; Harrison et al., 2020). However, these target gene mutations are only present in 15%–20% of patients, and targeting agents are ineffective in a small portion of patients with advanced LUAD (Mayekar and Bivona, 2017). Therefore, novel biomarkers and therapeutic targets are needed to predict prognosis and improve the survival of patients with LUAD.

The tumor microenvironment (TME) refers to the ecosystem surrounding the tumor, which includes immune cells, blood vessels, extracellular matrix, stromal cells, and signaling molecules (Anderson and Simon, 2020). Recent studies have shown that interactions between the tumor and TME play an important role in LUAD initiation, development, and progression (Hanahan and Coussens, 2012; Altorki et al., 2019). Studies elucidating the molecular and cellular biology of the TME have led to the development of novel immunotherapy strategies, including checkpoint blockade, adoptive cellular therapy, and cancer vaccinology (Waldman et al., 2020). Drugs targeting various components of the TME have been used in clinical trials and have demonstrated durable responses in patients with NSCLC (Gettinger et al., 2016; Herbst et al., 2018). Immune checkpoint blockades of programmed death-1 (PD-1) and its ligand, PD-L1, are the most effective treatments for LUAD, as they positively regulate T cell activation. As one of the most effective anti-PD-1 drugs, nivolumab has been shown to significantly improve the 5-year overall survival (OS) of patients with advanced NSCLC, compared with chemotherapy (Saka et al., 2021). However, the clinical efficacy of anti-PD-1 drugs has been reported in only 10% of patients with PD-L1-expressing tumors (Borghaei et al., 2015), and most patients with PD-L1^+^ tumors respond shortly.

Adoptive T cell (ATC) therapy, which involves the infusion of autologous or allogeneic T cells, is an efficient and promising cancer treatment approach. Allogenic hematopoietic stem cell transplantation was the first effective adoptive transfer approach used for the clinical treatment of leukemia, and the T cell graft-versus-tumor effect produced an improved prognosis (Weiden et al., 1979). Recently, a novel ATC therapy using autologous patient T cells redirected against specific antigens was shown to be an efficient treatment for blood cancers and has been approved for clinical applications (Munshi et al., 2021). However, the response and cure rates still require improvement, especially in the treatment of solid tumors. Owing to the suppression of T cell effector functions in the TME of solid tumors, the efficiency of chimeric antigen receptor T therapy in solid tumors is much lower than that in blood cancers. Moreover, the generation of adaptive immune responses in patients with cancer depends on the antigen-specific activation of naive T cells and the coordination of T cell signaling. Thus, regulators of T cell proliferation (TPRs) in solid tumors may be ideal targets for improving ATC immunotherapy.

In this study, we comprehensively evaluated the characteristics of TPRs in 526 patients with LUAD and identified three subgroups of TPRs associated with distinct immune infiltration patterns, prognoses, genomic features, and clinicopathological characteristics. We then established a TPR-related risk model to quantify T cell activation patterns in individuals. The model was shown to be a robust prognostic factor and predictive biomarker for the response to drugs in patients with LUAD.

## Materials and methods

### Data collection

A total of 1,883 patients with LUAD from six independent datasets were included in this study. TRP-related genes were extracted from the Gene Set Enrichment Analysis (GSEA) database (https://www.gsea-msigdb.org/gsea/msigdb/cards/GOBP_ACTIVATED_T_CELL_PROLIFERATION.html) and Legut et al. (2022) report (Supplementary Table S1). Gene expression data, gene mutation data, and LUAD clinical profiles from The Cancer Genome Atlas (TCGA) were acquired from the XENA database (https://xena.ucsc.edu/). The gene expression matrix and corresponding survival files from GSE68465, GSE50081, and GSE72094, based on the Affymetrix Human Genome platform, were downloaded from the Gene Expression Omnibus database. Gene expression data and survival profiles for the validated cohort were extracted from the GEO database (GSE42127 and GSE36471 datasets). The Affy package was used to perform a background adjustment among these datasets (Gautier et al., 2004). According to the empirical Bayes framework using the sva package, we adjusted and removed batch effects between the different expression profiles, which were subsequently merged to form a mixed cohort for further analyses. To prevent influencing the accuracy of patient survival predictions, this study did not include patients without prognostic data. The baseline information of the patients with LUAD is shown in Table 1.

**TABLE 1:** Clinical baseline features of the LUAD patients in three databases.

	GSE36471		GSE42127		TCGA-LUAD		*p*-value
	High	Low	High	Low	High	Low	
	(*N* = 58)	(*N* = 58)	(*N* = 88)	(*N* = 88)	(*N* = 250)	(*N* = 250)	
TPS							
Subtype 1	37 (63.8%)	8 (13.8%)	15 (17.0%)	23 (26.1%)	46 (18.4%)	161 (64.4%)	<0.001
Subtype 2	11 (19.0%)	31 (53.4%)	23 (26.1%)	50 (56.8%)	99 (39.6%)	38 (15.2%)	
Subtype 3	10 (17.2%)	19 (32.8%)	50 (56.8%)	15 (17.0%)	105 (42.0%)	51 (20.4%)	
Event							
Death	36 (62.1%)	30 (51.7%)	41 (46.6%)	23 (26.1%)	116 (46.4%)	66 (26.4%)	<0.001
Alive	22 (37.9%)	27 (46.6%)	47 (53.4%)	65 (73.9%)	134 (53.6%)	184 (73.6%)	
Missing	0 (0%)	1 (1.7%)	0 (0%)	0 (0%)	0 (0%)	0 (0%)	

### Unsupervised consensus clustering of T cell proliferations

The ConsensusClusterPlus package was used to perform consensus analysis. LUAD samples were divided into three clusters based on significantly differential TPR-associated gene expression levels (false discovery rate <0.05 and |fold change| > 0.5) (Wilkerson and Hayes, 2010). Among the different k-means clustering results (*k* = 2–7), three groups (*k* = 3) demonstrated the most stable discrimination.

### Survival and clinical analysis

OS was evaluated in each group using the Kaplan-Meier (KM) method and compared among groups using the log-rank test. The chi-square test was used to compare differences between groups. The threshold for statistical significance was defined as a *p*-value less than 0.05.

### Pathway enrichment analysis

To exploit the potential processes between TPR group, we utilized the limma package [PMID: 25605792] to perform the differential expression analysis between different TPR and risk group. Firstly, the differential expression genes (DEGs) were obtained between TPRs group and risk group by differential expression analysis using limma package. Then, we screened the DEGs at certain condition (log2FC > 1 and adjust *p*-value < 0.05). Final, those DEGs were enrolled to performed next step enrichment analysis. The GSEA analysis of different groups from two independent cohorts was performed (Powers et al., 2018). The profiles extracted from the GSEA database (http://www.gsea-msigdb.org/gsea/downloads.jsp; project: h.all.v7.5.1. symbols.gmt) were analyzed using a reference gene set. Kyoto Encyclopedia of Genes and Genomes (KEGG) and Gene Ontology (GO) enrichment analyses were conducted for the different groups using the clusterProfiler package.

### Immune infiltration analysis

The MCP-counter method can be used to infer the immune and stromal cell composition of heterogeneous tissue (Aran et al., 2017; Petitprez et al., 2020). The IOBR package was used to assess T cell infiltration *via* the MCP-counter method (Zeng et al., 2021). The ESTIMATE algorithm was used to evaluate the immune score and stromal score in different groups *via* the IOBR package (Yoshihara et al., 2013). Tumor immune dysfunction and exclusion (TIDE) scores were calculated using the TIDE online database (http://tide.dfci.harvard.edu/) (Fu et al., 2020). The T cell exhaust score (gene set come from IOBR package) and T cell activation score (gene set come from: http://cis.hku.hk/TISIDB/index.php) were calculated by ssGSEA algorithm.

### Construction and validation of the least absolute shrinkage and selection operator-Cox regression model

We first identified TPR-related genes that were significantly differentially expressed between LUAD tissues and normal lung tissues. Univariate Cox regression analysis was used to determine the OS associated with TPR-related genes (*p* < 0.05). Finally, LASSO-Cox regression analysis was performed. Nine key TPRs in LUAD were identified and used to construct a TPR-related risk model for LUAD. The risk score for each patient was calculated using the following formula: risk score = −0.004491532* AGER (gene expression level) + (−0.077486959* CYP27A1) + 0.113176339* CDK1 + (−0.0453135648* CADM1) + 0.342268624* FADD+ 0.133393571* ADA +0.024214523* LTBR + (−0.022959715* FYN) + (−0.199900841* CRTAM).

### Predictive efficacy of the model

Time-dependent receiver operating characteristic (ROC) curves were used to assess 1-, 3-, and 5-year OS. The predictive efficacy of the risk model was determined by assessing the area under the curve (AUC).

### Correlations between clinical characteristics and T cell proliferation signature

Correlations between clinical features (age, sex, stage, and TNM stage) and the TPR signature were evaluated using the chi-square test. The TPR signature was then differentiated into subgroups based on these clinical characteristics. Univariate and multivariate Cox regression analyses were used to identify independent indicators of patient survival.

### Nomogram construction and assessment

Univariate Cox regression analysis was performed to screen for significant factors (*p* < 0.05), which were subjected to further multivariate analysis and used for nomogram construction. The concordance index (C-index) was used to compare the predictive ability of the nomogram and the clinical features. Calibration plots were constructed to determine the fitting efficiency between the nomogram-predicted OS and actual OS. Decision curve analysis was used to assess the threshold expectation range of the nomogram in association with clinical characteristics.

### Relationship between chemoresistance and T cell proliferation signature

The half-maximal inhibitory concentration (IC50) of FDA-approved drugs (rapamycin, cisplatin, paclitaxel, bortezomib, elesclomol, tipiifarnib, nilotinib, and doxorubicin) was determined for each TCGA-LUAD patient using the pRRophetic package. The IC50 was used to differentiate between high and low risk scores.

### RNA extraction and quantitative PCR

Total RNA was extracted from U87 cells using TRIzol reagent (Invitrogen, Carlsbad, CA, United States), and reverse transcription was performed using the PrimeScript™ RT Reagent Kit (Takara, Dalian, China). cDNA was subjected to RT-qPCR using the SYBR Green Real-Time PCR Kit (Takara, Dalian, China). Relative mRNA expression were normalized to that of β-actin. The relative expression were calculated using the 2^−ΔΔCT^ method.

### Statistical analysis

R (version 4.0.2) was used for statistical analysis. A *p*-value < 0.05 was regarded as indicative of a statistically significant difference. Comparisons between two groups were conducted using Student’s *t*-test or the Kruskal-Wallis H test, and comparisons among three or more groups were conducted using the Wilcoxon signed-rank test. Clinicopathological data for TCGA-LUAD patients grouped by the TPR model were analyzed using the chi-square test, and the log-rank test was used for survival analysis.

## Results

### Characterization of T cell proliferation patterns

**FIGURE 1:**
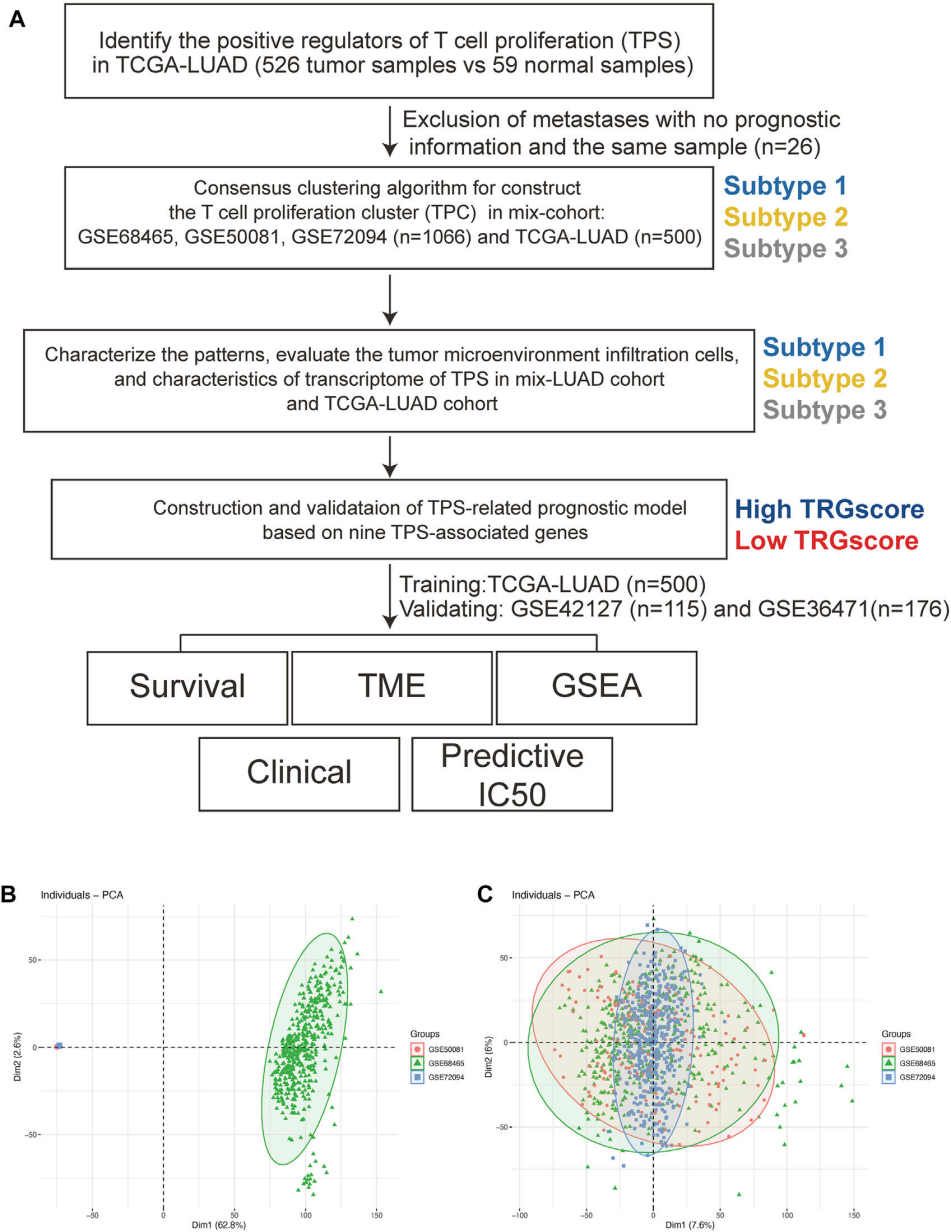
The workflow of this study. **(A)** The workflow chart of this study. **(B,C)** Principal component analyses before **(B)** and after **(C)** batch removal.

The TPR infiltration patterns were systematically evaluated, and a TPR signature was constructed (Figure 1A). We integrated 1,066 LUAD samples from the same GEO platform and constructed T cell proliferation clusters (TPCs) in the mixed cohort (GSE68465, GSE50081, and GSE72094). Principal component analysis revealed changes in the sample distribution before and after integration (Figures 1B,C).

**FIGURE 2:**
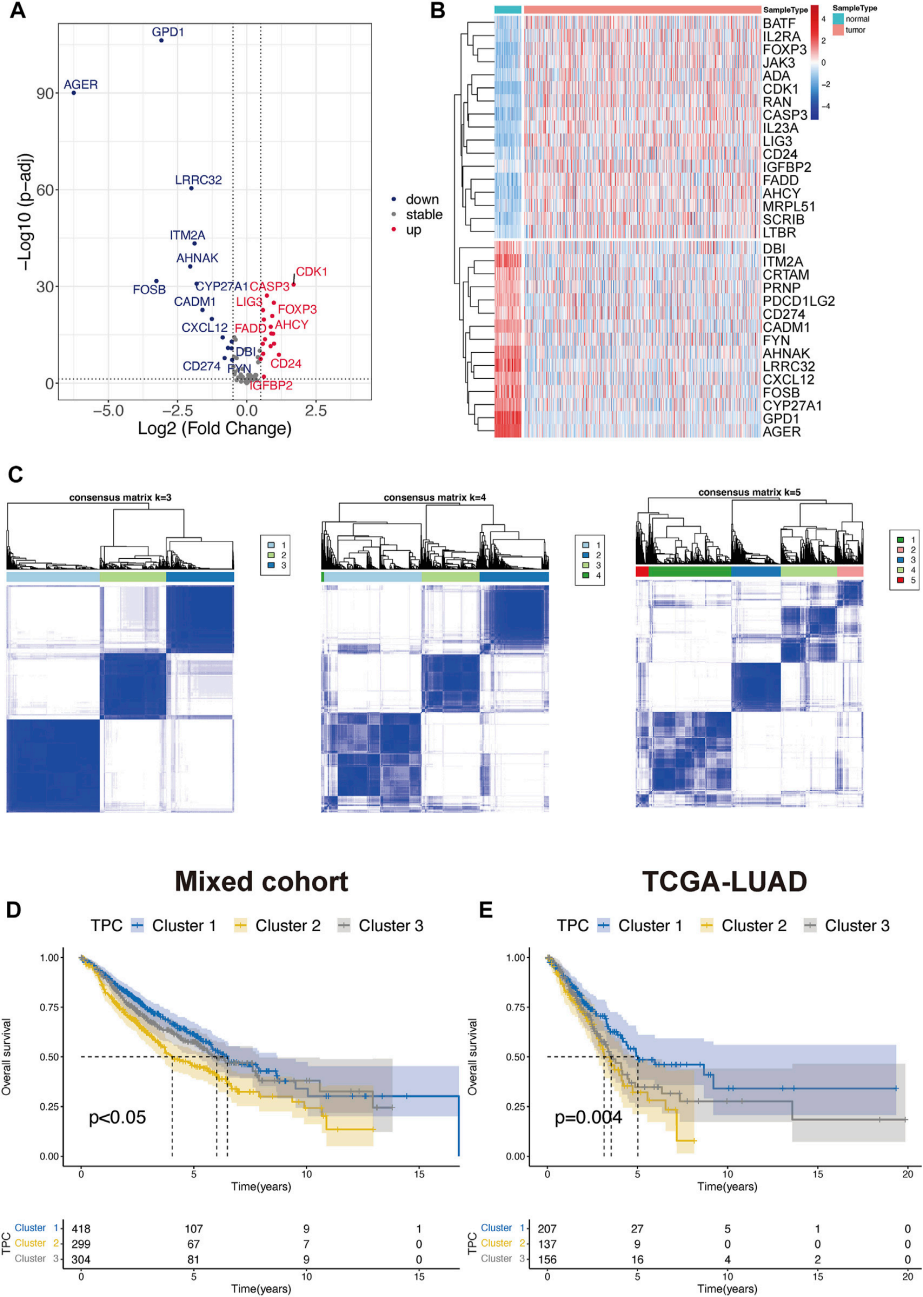
Identify the T cell proliferation cluster (TPC). **(A,B)** Volcano diagram and heatmap of the positive drive of T cell proliferation that depicts the abnormal differentially express pattern in lung adenocarcinoma and normal tissue samples. Blue dots: down-regulation, grey dots: none significance differential genes, and red dots: up-regulation. **(C)** Unsupervised hierarchical analyses of the differential expression patterns of these T cell-associated genes in mix-cohort (k-means = 3–5). **(D,E)** Comparison of overall survival between TPC by using Kaplan-Meier survival curves in mix-cohort **(D)** and TCGA-LUAD **(E)**.

To determine the optimal cluster number, we identified differentially expressed TPRs between LUAD tumors and normal lung tissues in TCGA-LUAD cohort (Figures 2A,B). Next, we evaluated the clustering stability using the ConsensusClusterPlus package, which indicated the existence of three powerful TPCs in both the mixed cohort and TCGA-LUAD cohort (Figure 2C). In addition, the KM survival curves revealed that the three main TPCs in the mixed and TCGA-LUAD cohort exhibited significant differences (log-rank test, *p* < 0.05; Figure 2D–E). In particular, cluster 2 was associated with worse survival outcomes than the other clusters.

**FIGURE 3:**
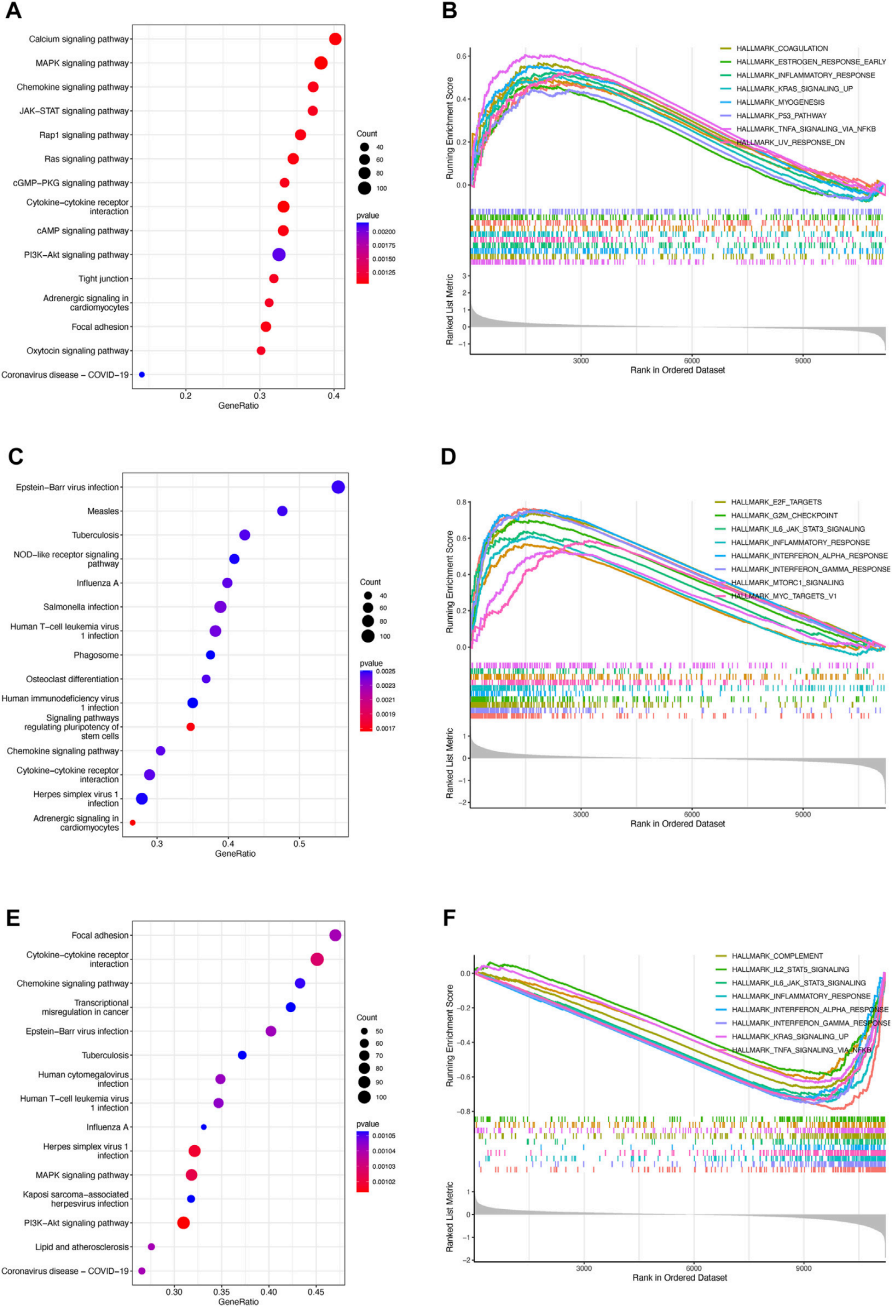
Enrichment analysis of TPRs **(A–F)**. Top 5 KEGG enriched gene pathway-related catalogs and top 10 GSEA of tumor-associated items in cluster 1 **(A,B)**, cluster 2 **(C,D)**, and cluster 3 **(E,F)**.

To exploit the potential processes between TPR group, we performed the differential analysis by Limma package. We explored the biological processes associated with the three TPR clusters by using the clusterProfiler package to perform KEGG pathway enrichment analysis and GSEA in the mixed cohort. Cluster 1 was markedly enriched in carcinogenic pathways, such as the cAMP signaling pathway, WNT signaling pathway, KRAS signaling pathway, and P53 pathway (Figures 3A,B; Supplementary Table S2). Cluster 2 exhibited enrichment in carcinogenic pathways (PI3K-AKT signaling pathway, MAPK signaling pathway, and IL6-JAK-STAT3 signaling pathway) but also exhibited robust positive correlations with biological processes associated with immune activation, including cytokine-cytokine receptor interaction, interferon-gamma response, and inflammatory response (Figures 3C,D). Interestingly, cluster 3 annotations included pathways that were negatively associated with cluster 2 (Figures 3E,F).

### Characteristics of T cell proliferation patterns in the tumor immune microenvironment

**FIGURE 4:**
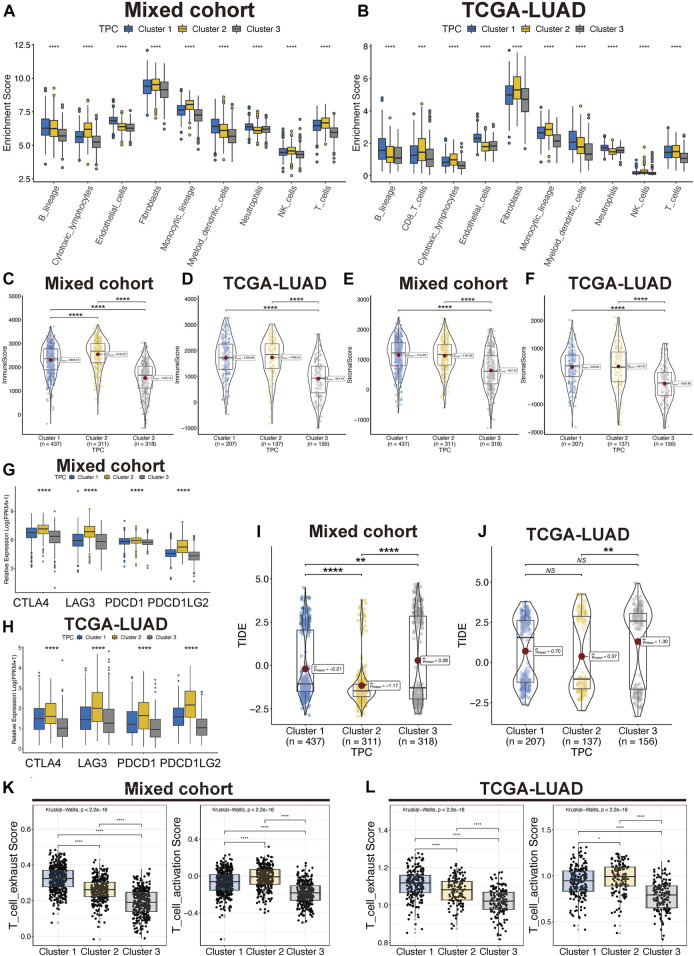
Tumor immune microenvironment of TPC. **(A,B)** Distribution of T cell in lung cancer and TME in mix-cohort **(A)** and TCGA-LUAD **(B)**. **(C–F)** ESTIMATE tumor purity algorithm was used to calculate the immune score and the stromal score of three TPC patients in the mix-cohort **(C,E)** and TCGA-LUAD **(D,F)** cohort. **(G,H)** Abnormal expression of immune checkpoint markers between TPC in mix-cohort **(G)** and TCGA-LUAD cohort **(H)**. **(I,J)** TIDE score of mix-cohort **(I)** and TCGA-LUAD **(J)** between TPC group. **(K,L)** T cell activation/exhaust score of mix-cohort **(K)** and TCGA-LUAD **(L)** between TPC group.

To identify correlations between TPR patterns and the TIME, we calculated the degree of infiltration of different types of immune and stromal cells by using single-sample GSEA to analyze the TPCs. The findings were consistent with the results shown in Figure 3, indicating that the degree of infiltration of the TPCs decreased in the following order in the mixed cohort and TCGA-LUAD cohort (Figures 4A,B): cluster 2 > cluster 1 > cluster 3. However, cluster 2, which had the highest degree of T cell infiltration and the strongest association with immune-related response pathways, was not associated with a corresponding survival advantage. Therefore, we evaluated the immune and stromal scores of the TPCs. In the mixed cohort and TCGA-LUAD cohort, cluster 2 had the highest scores, and cluster 1 had the lowest scores (Figures 4C–F; cluster 2 > cluster 1 > cluster 3). Although cluster 2 exhibited CD4/8 + T cell activation, this cluster also exhibited stromal cell activation, which exerts an immunosuppressive effect. Therefore, for these patients, immunosuppressive therapy may be suitable as a first-line treatment.

In addition, we examined the expression of four immune checkpoint genes (*PDCD1*, *PDCD1LG2*, *CTLA4*, and *LAG3*), which are related to immune blockage. The expression of these genes in the mixed cohort (Figure 4G) and TCGA-LUAD cohort (Figure 4H; Supplementary Table S3) decreased in the following order: cluster 2 > cluster 1 > cluster 3. Patients in cluster 2 had the lowest TIDE scores in both the TCGA-LUAD and mixed cohorts, suggesting that these patients are most likely to benefit from immunotherapy (Figures 4I,J). In addition, Cluster 2 have moderate exhaust and activation score of T cell (Figures 4K,L). Cluster 3 was characterized as an immune-desert phenotype. Cluster 2, which featured robust T cell immune filtration and a high stromal score, was characterized as an immune-inflamed phenotype. Cluster 1, which featured a moderate immune score and moderate immune infiltration with T cells, was characterized as an intermediate phenotype.

### Construction and validation of T cell proliferation-related risk model

**FIGURE 5:**
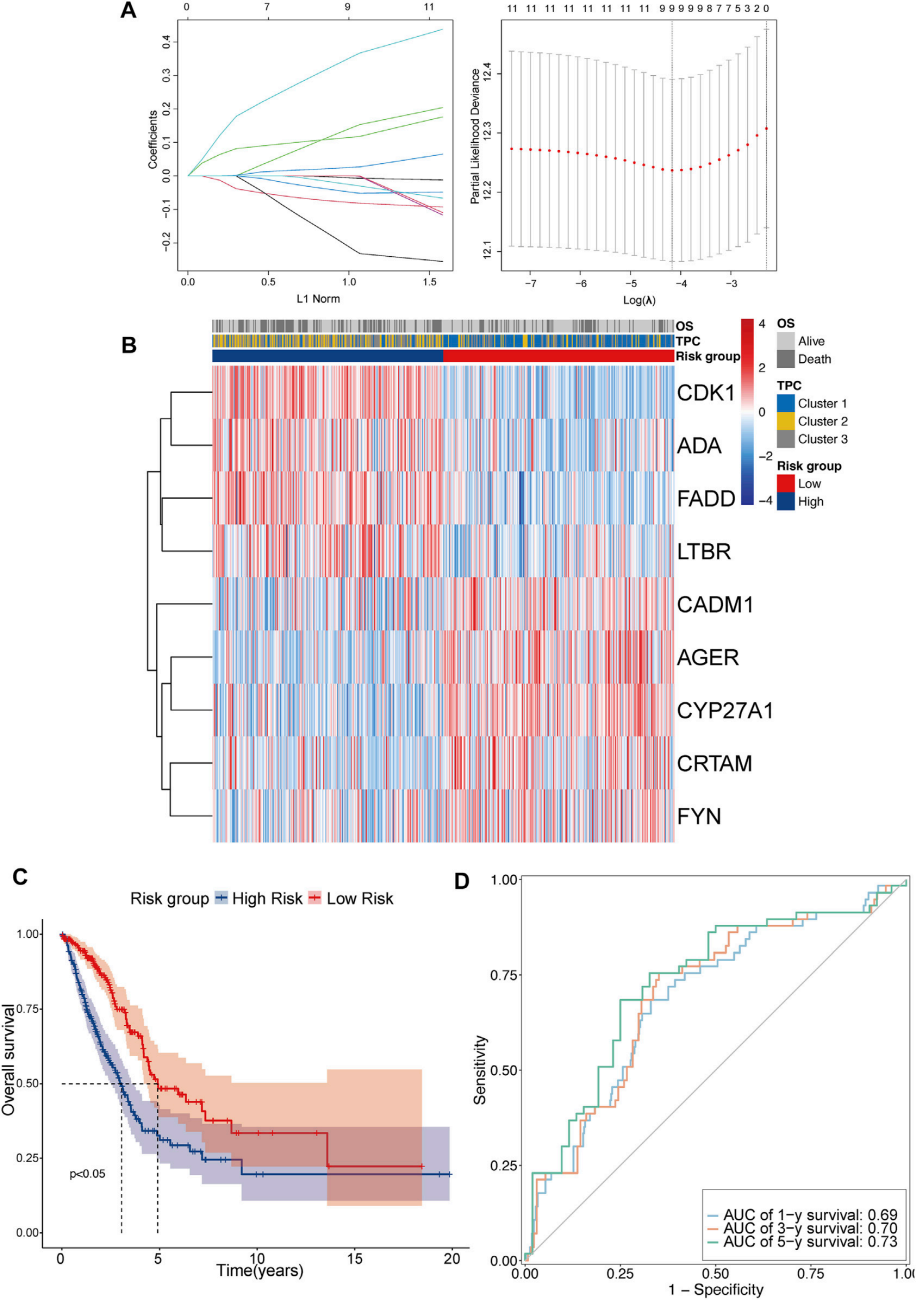
Construct the TPR-related risk model in TCGA-LUAD. **(A,B)** Lasso-Cox regression model analysis results and heatmap of T cell proliferation prognostic model signature: **(A)** lasso regression analysis (left panel); partial likelihood deviance for the lasso regression (right panel); dotted line: lambda.min (left) and lambda.se (right); **(B)** heatmap between high and low-risk scores and clinical parameters. **(C)** KM curves showing OS in patients with risk group; blue line: high-risk score (*n* = 250) and red line: low-risk score (*n* = 250). **(D)** Receiver operating characteristic (ROC) curve of 1 (light blue), 3 (orange), and 5 years (green).

TPRs play a critical role in the regulation of different T cell functions. As TPR pattern prediction in individuals is not a suitable analysis method, TPCs were identified in the population. To account for the individual heterogeneity and complexity of TPR patterns, we aimed to construct a TPR-associated risk model to quantify the TPR patterns of individuals with LUAD. To illustrate TPR patterns in transcriptomic data, 11 TPRs were selected using univariate Cox regression analysis (Supplementary Table S4). LASSO-Cox regression was used to identify nine candidate prognostic genes, which were then used to establish the risk score (Figure 5A). The coefficients of each TPR are shown in Supplementary Table S3. The heatmap depicts the transcriptome characteristics associated with the risk score and the distribution of risk scores among the TPCs (Figure 5B). KM survival curves showed that the OS of LUAD patients with low-risk scores was better than that of those with high-risk scores (Figure 5C). The AUC for the time-dependent ROC curve, which was used to evaluate the predictive efficacy of the prognostic model, was 0.69 for 1-year survival, 0.70 for 3-year survival, and 0.73 for 5-year survival (Figure 5D).

**FIGURE 6:**
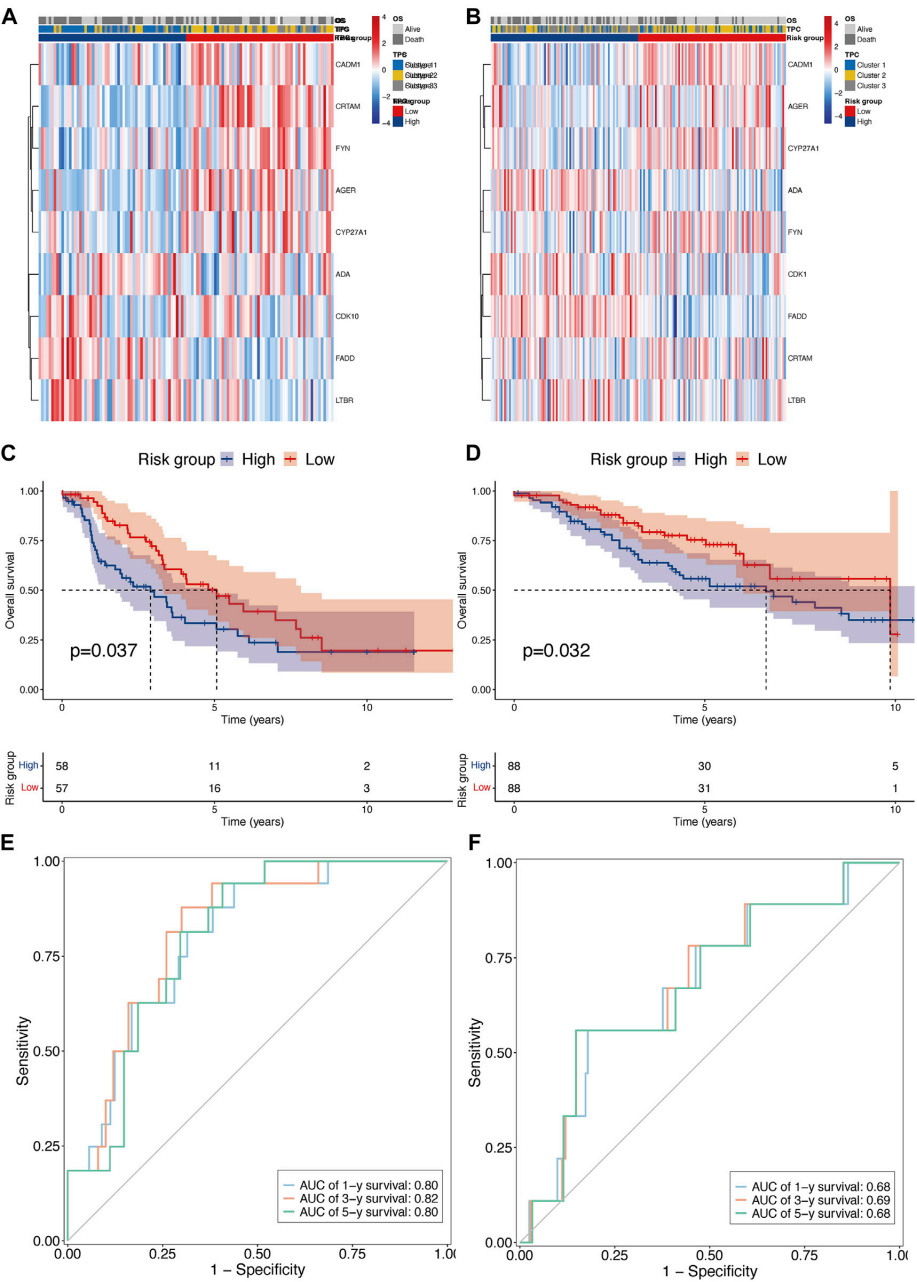
External validation of TPR signature in GSE42127 and GSE36471 cohorts. **(A,B)** Heatmap of nine TPR signature in external validate cohort (GSE36471 *n* = 115: left, and GSE42127 *n* = 176: right). **(C,D)** KM curves showing OS in patients with TPR signature (Left: GSE36471, and right: GSE42127). **(E,F)** AUC curves of 1 (light blue), 3 (orange), and 5 years (green) in both external cohorts (Left: GSE36471, and right: GSE42127).

To confirm the reproducibility and stability of the TPR signature, two independent LUAD cohorts acquired from the GEO database were used for external validation. The transcriptome features of the two validation sets were consistent with those of the training set (Figures 6A,B). KM survival analysis also indicated that patients in the validation cohorts with high-risk scores were associated with a poor OS, compared with those with low-risk scores (Figures 6C,D). Similarly, the AUCs for GSE42127 (Figure 6E; 1-year AUC = 0.80, 3-year AUC = 0.82, 5-year AUC = 0.80) and GSE36471 (Figure 6F; 1-year AUC = 0.68, 3-year AUC = 0.69, 5-year AUC = 0.68) indicated that the TPR signature exhibited excellent performance when used to predict the OS of LUAD patients.

### Tumor mutation characteristics of T cell proliferation clusters

**FIGURE 7:**
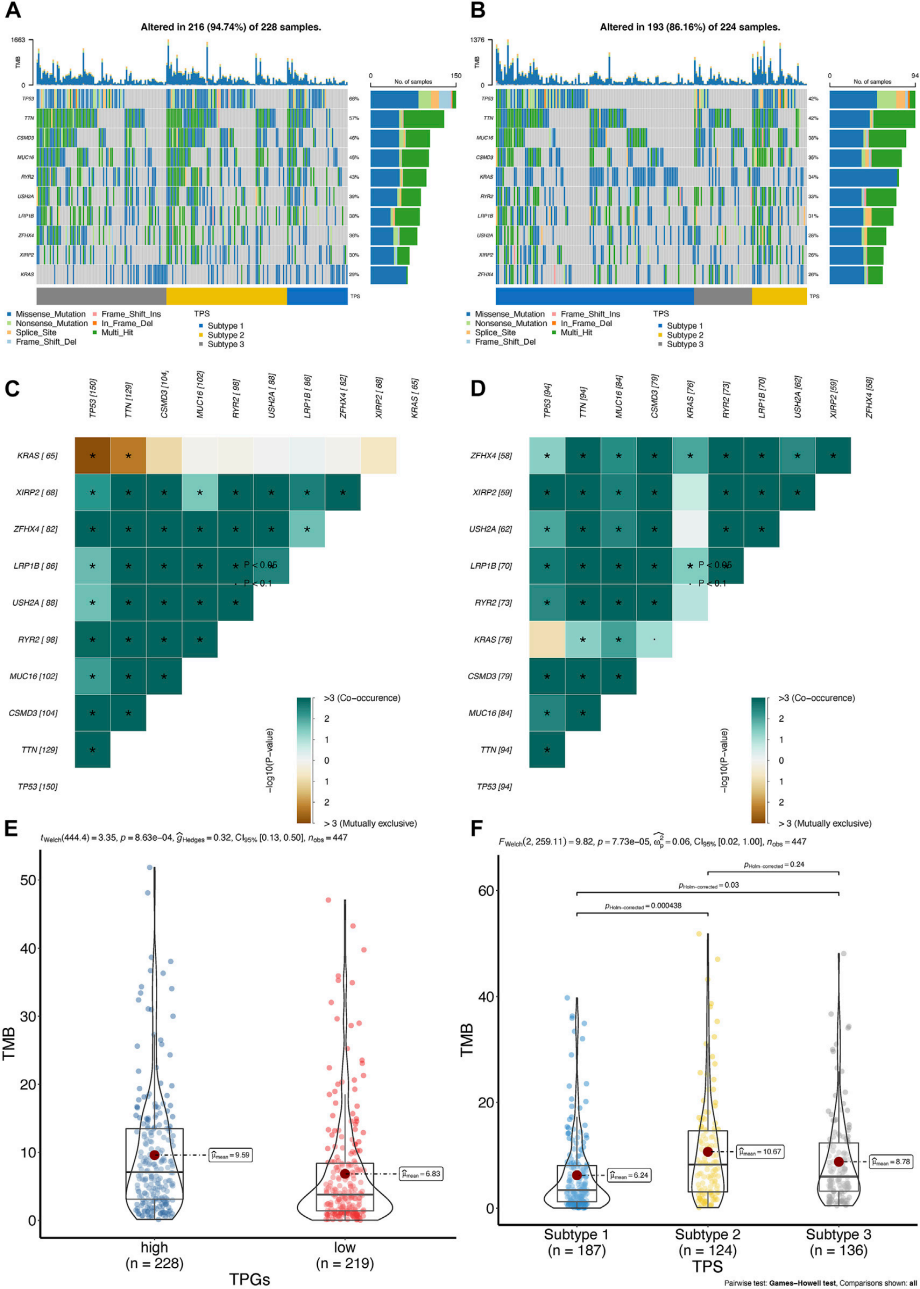
The landscape of somatic mutation between high- and low-risk group in TCGA-LUAD cohort. **(A,B)** Oncoplot of genes with highest counts of variants between high- **(A)**, *n* = 250 and low-risk score **(B)**, *n* = 250 in TCGA-LUAD cohort. **(C,D)** Significant exclusive or co-occurrence top 10 mutation gene sets are indicated in the high-risk score **(C)** and low-risk score **(D)** in TCGA-LUAD. **(E,F)** Distribution of tumor mutation burden in TPR signature **(E)** and TPC **(F)** group patients; TMB, tumor mutation burden.

To investigate whether the distinct T cell prognostic phenotypes were determined by genetic events, we conducted an integrative analysis of the mutation data. We first explored the quantity and quality of somatic mutations in the high- and low-risk groups of the TCGA-LUAD cohort. As depicted in Figures 7A,B, the frequency of TP53 mutations was significantly higher in the high-risk group than in the low-risk group.

Mutually exclusive or co-occurring gene mutations are frequently observed in cancer patients (Kang et al., 2008; Kim et al., 2017). Detecting such mutation patterns is critical for identifying novel cancer signaling pathways and developing potential therapeutic strategies. As shown by the differences among the top 10 genes in the heatmap, TP53 and KRAS mutations exhibited mutual exclusivity (*p* < 0.05) in the high-risk group, but not in the low-risk group (Figure 7C). In addition, co-occurring mutations in TTN and KRAS were identified in the low-risk group, whereas mutual exclusivity was observed in the high-risk group (Figure 7D).

In patients with LUAD, tumor mutation burden (TMB) has been regarded as an independent predictor of immunotherapy success (Goodman et al., 2017; Hellmann et al., 2018). As shown in the violin plot, patients belonging to the low-risk group or cluster 2 had a higher TMB, indicating that they may respond to PD-1/PD-L1 blockade therapy (Figures 7E,F). Therefore, we further assessed the differences in the TME among the TPR signature groups.

### Characteristics of T cell proliferation-related risk model

**FIGURE 8:**
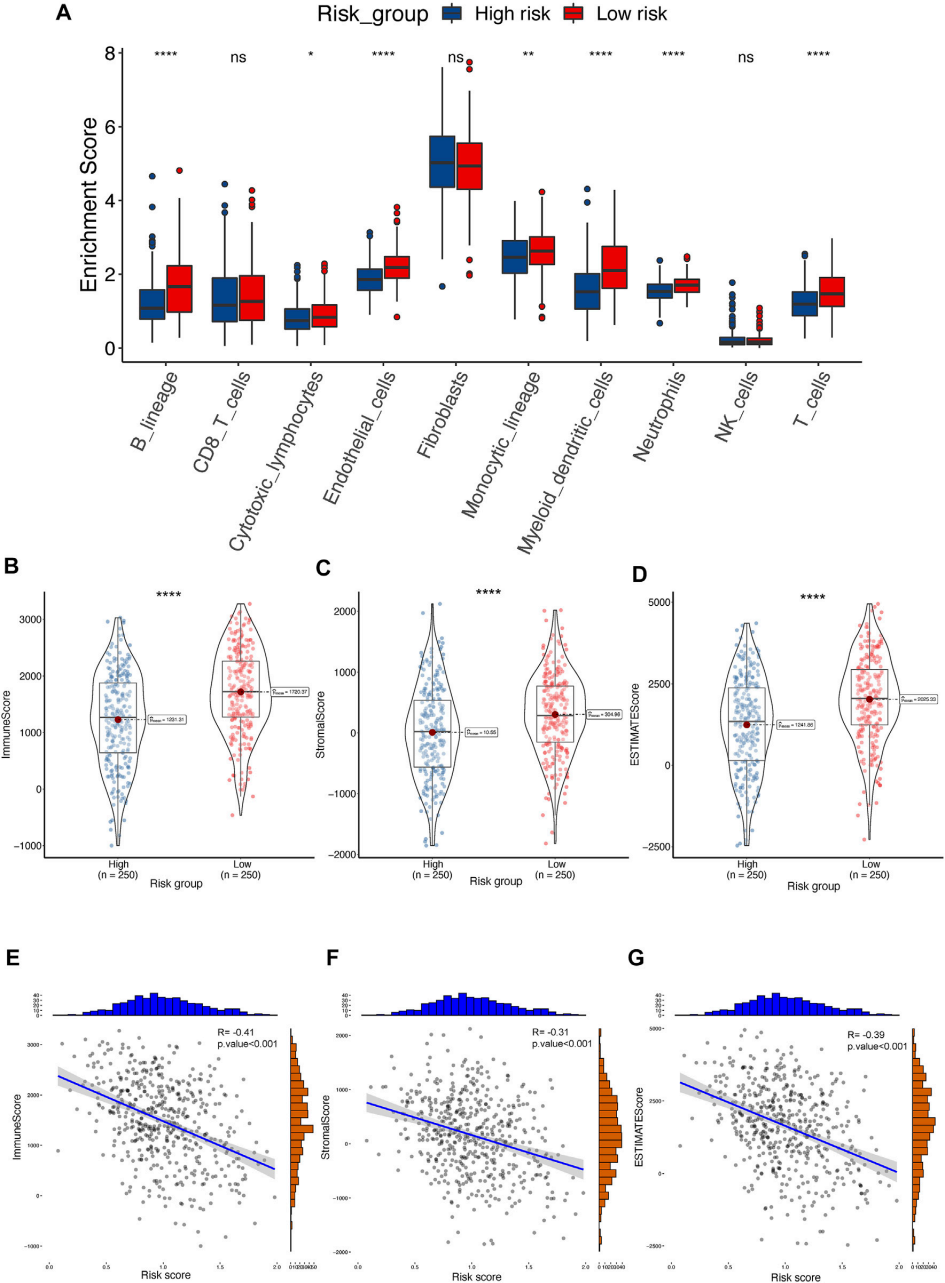
The landscape of TME between high- and low-risk groups in TCGA-LUAD cohort. **(A)** Distribution of T cell between TPR signature in TCGA-LUAD. **(B–D)** ESTIMATE tumor purity algorithm was utilized to calculate an immune score **(B)**, stromal score **(C)**, and tumor purity **(D)** of risk-related patients in the TCGA-LUAD cohort. **(E–G)** Pearson correlation analyses between risk score and immune score **(E)**, stromal score **(F)**, and estimate score **(G)** in the TCGA-LUAD cohort.

We quantified immune cell infiltration using the MCP-counter algorithm to further investigate the association between TPRs and the TME. Consistent with the TMB analysis results, the low-risk group exhibited a higher degree of T cell infiltration than the high-risk group (Figure 8A). Moreover, the ESTIMATE algorithm results revealed a greater elevation in the immune score, stromal score, and estimate score in the low-risk group (Figures 8B–D). Similarly, the Pearson correlation coefficients also indicated that risk was negatively associated with the immune-associated scores (Figures 8E–G; immune score R = −0.41, stromal score R = −0.31, and estimate score R = −0.39; *p* < 0.001).

**FIGURE 9:**
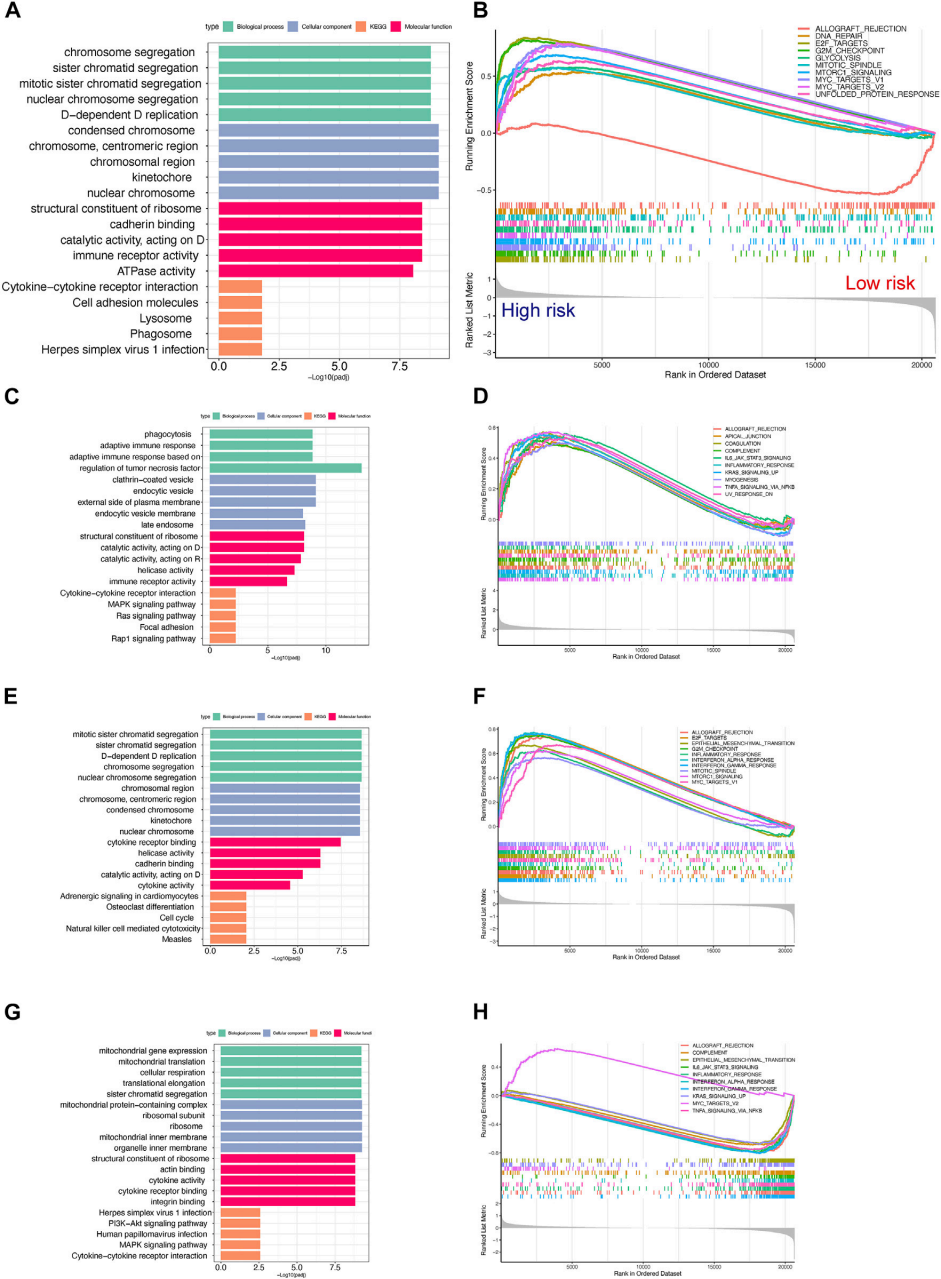
Gene set enrichment analysis for high- and low-risk scores in the TCGA-LUAD cohort. **(A,B)** Top 5 GO and KEGG enriched gene pathway-related catalogs **(A)** and top 10 GSEA **(B)** of tumor-related items between high- and low-risk scores in TCGA-LUAD **(C,D)**. Cluster 1 in GO/KEGG analysis **(C)** and GSEA **(D)** results in the TCGA-LUAD cohort (*n* = 207) **(E,F)**. Cluster 2 in GO/KEGG analysis **(E)** and GSEA **(F)** results in the TCGA-LUAD cohort (*n* = 137). **(G,H)** Cluster 3 in GO/KEGG analysis **(G)** and GSEA **(H)** results in the TCGA-LUAD cohort (*n* = 156).

To further explore the potential differences in biological functions between the TPR-associated groups, GO and KEGG enrichment analyses of hallmark pathways in the high-risk and low-risk groups were performed. Chromatid segregation-related pathways and cytokine-cytokine receptor interaction pathways were significantly enriched (Figure 9A; Supplementary Table S5). Similar to cluster 3, the low-risk group displayed more enrichment in immune-related functions than the high-risk group, including the interferon-associated response, inflammatory response, and IL2/STAT5 signaling pathway (Figure 9B). Like the previous TPC results, the enrichment results for TCGA-LUAD cohort were consistent with those for the mixed cohort (Figures 9C–H).

### Subgroup overall survival analysis

**FIGURE 10:**
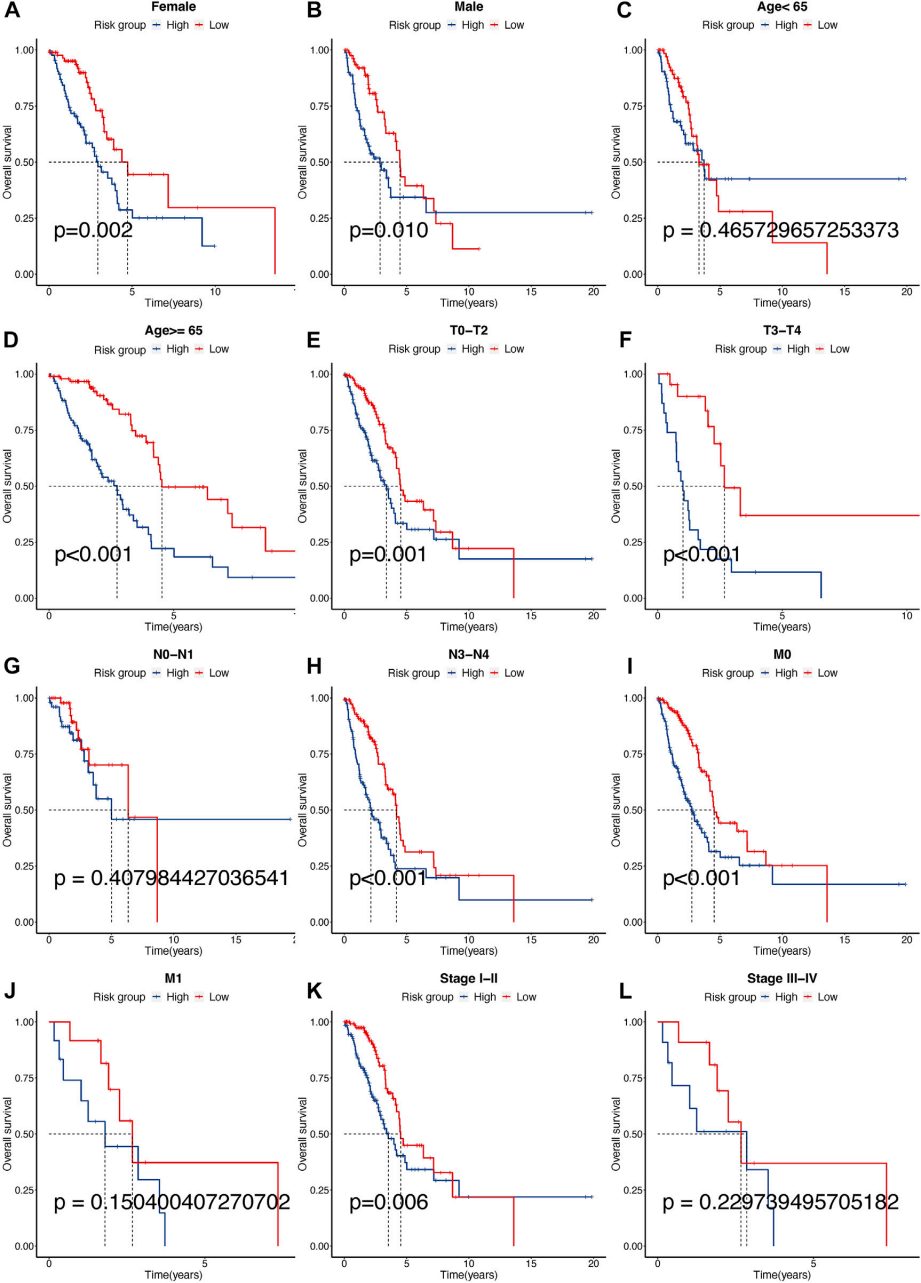
Different clinical sub-group survival analyses between high- and low-risk groups in the TCGA-LUAD cohort. **(A–L)** KM analysis between gender (Female: a, Male: b), age (<65: c, ≥65: d), T (TX-T2: e, T3-T4: f), N (N0-N1: g, N2-N3: h), M (M0: i, M1: j), and stage (I-II: k, III-IV: l) for high- and low-risk scores in TCGA-LUAD cohort.

Clinical subgroup OS analysis demonstrated that the TPR signature was suitable for predicting survival in older (≥65 years), N2-N3 stage, M0 stage, or stage I-II LUAD patients. Among these patients, high risk was correlated with a notably poor OS. Sex and T stage did not affect the TPR model. Furthermore, a statistical difference (log-rank test) in OS between the high-risk group and the low-risk group was not observed in younger (<65 years), N0-N1 stage, M1 stage, or stage III-IV patients (Figures 10A–L).

### Prediction of drug sensitivity

**FIGURE 11:**
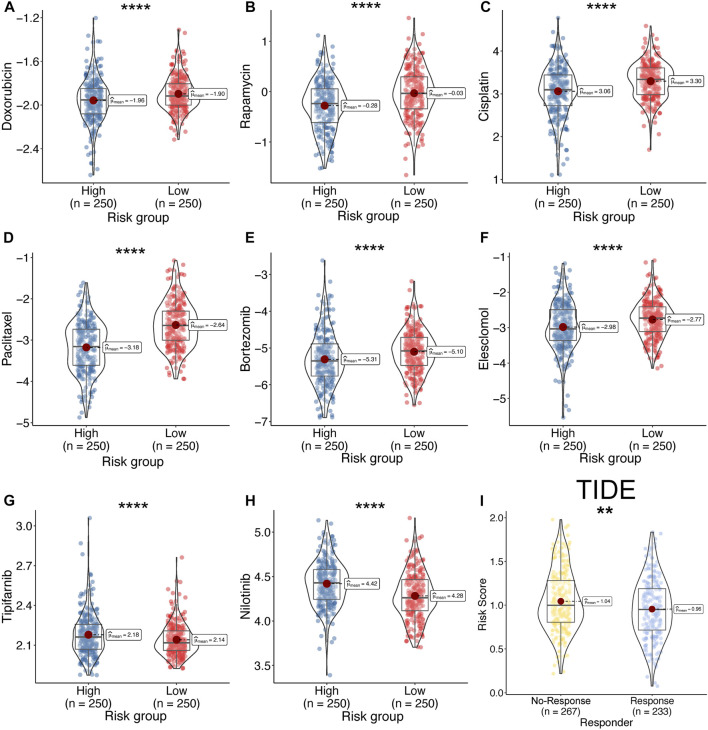
Drug sensitivity between high- and low-risk. **(A–H)** Calculate the half-maximal inhibitory concentration of FDA-approved drugs in the risk model, including, Doxorubicin **(A)**, Rapamycin **(B)**, Cisplatin **(C)**, Paclitaxel **(D)**, Bortezomib **(E)**, Elesclomol **(F)**, Tipifarnib **(G)**, and Nilotinib **(H)**. **(I)** Response rate between high- and low-risk group by TIDE algorithm in TCGA-LUAD cohort.

The predictive IC50 was calculated using the pRRophetic package. The high-risk group exhibited more sensitivity to doxorubicin (Figure 11A, High_median = −1.96 vs. Low_median = −1.90, *p* < 0.001), rapamycin (Figure 11B, High_median = −0.28 vs. Low_median = −0.03, *p* < 0.001), cisplatin (Figure 11C, High_median = −3.06 vs. Low_median = −3.30, *p* < 0.001), paclitaxel (Figure 11D, High_median = −3.18 vs. Low_median = −2.64, *p* < 0.001), bortezomib (Figure 11E, High_median = −5.31 vs. Low_median = −5.10, *p* < 0.001), and elesclomol (Figure 11F, High_median = −2.98 vs. Low_median = −2.77, *p* < 0.001). The low-risk group exhibited more sensitivity to tipifarnib (Figure 11G, High_median = 2.18 vs. Low_median = −2.14, *p* < 0.001) and nilotinib (Figure 11H, High_median = 4.42 vs. Low_median = −4.28, *p* < 0.001). In addition, we predicted the response rate to immunotherapy in the TCGA-LUAD cohort using the TIDE algorithm. These results indicated that immunotherapy may be more suitable for patients with a lower risk score (Figure 11I).

### Nomogram construction and assessment

**FIGURE 12:**
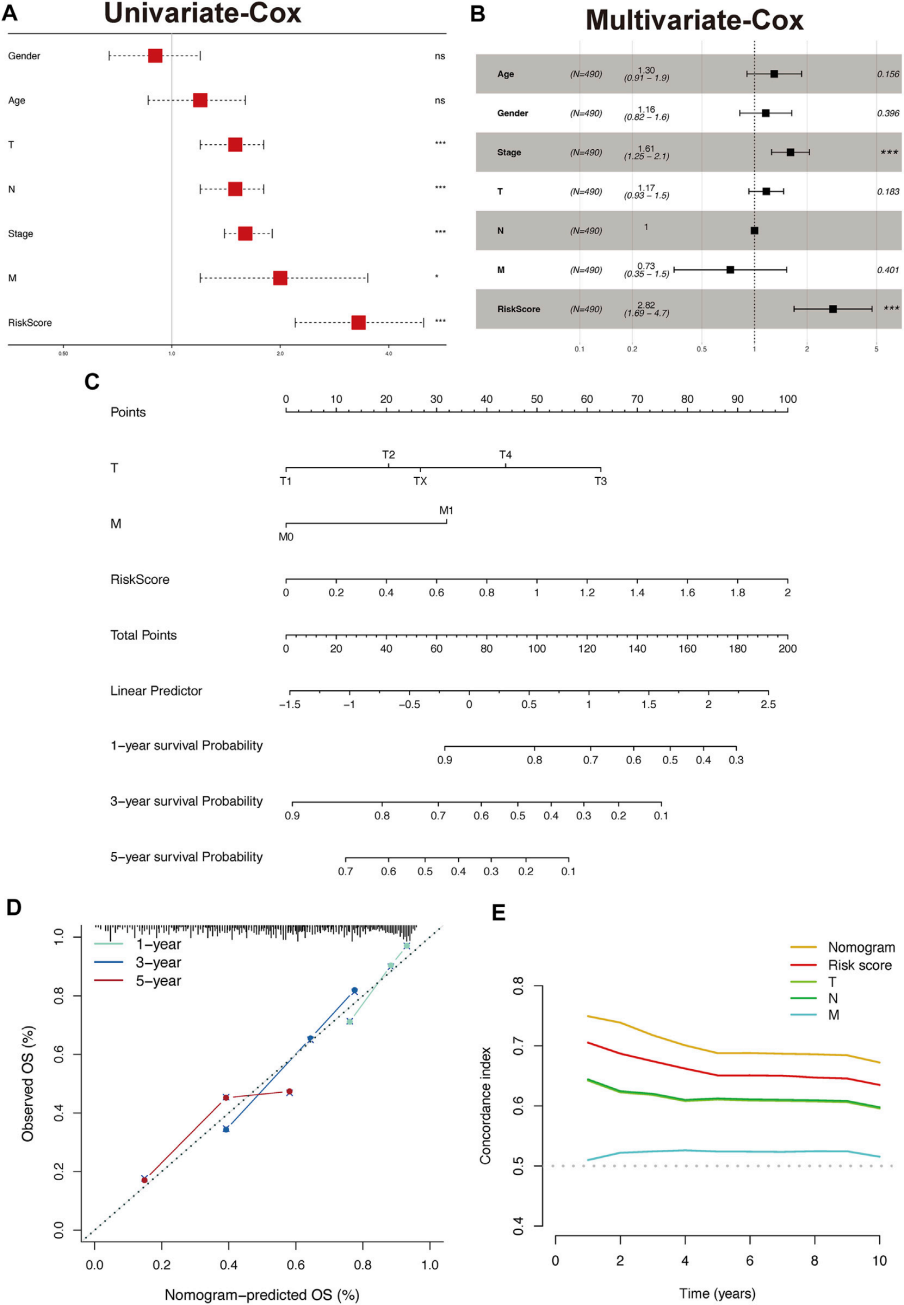
Construct and validate the nomogram model. **(A,B)** Univariate **(A)** and multivariate **(B)** analyses were performed using Cox regression of the TCGA-LUAD cohort. **(C)** Nomogram based on TPR signature and Stage in TCGA-LUAD. **(D)** Calibration of a nomogram predicting 1-, 3-, and 5-year OS. **(E)** Distributions of concordance index values in a nomogram and relevant clinical data.

The results of the univariate analysis indicated that TPR signature, T stage, N stage, and M stage and stage were associated with OS (Figure 12A, *p* < 0.001). To exclude the interference of other phenotypes to the prognosis, we perform a multifactorial cox analysis of TPR signature in the TCGA-LUAD cohort. As shown in Figure 12B, the TPR signature was identified as independent prognostic variables associated with OS. Stage and TPR signature were included in a nomogram model established for predicting OS in clinical settings (Figure 12C). Calibration and C-index curves were used to assess the agreement between the actual prognosis value and the value predicted by the nomogram. The calibration curves for the 1-, 3-, and 5-year survival rates exhibited a close fit with the nomogram values (Figure 12D). According to the C-index curves, in terms of predictive ability, the nomogram and clinical data performed in the following order: nomogram > TPR signature > T/N stage > M stage (Figure 12E).

### Validation of the expression of T cell proliferation signature in lung adenocarcinoma cell lines

**FIGURE 13:**
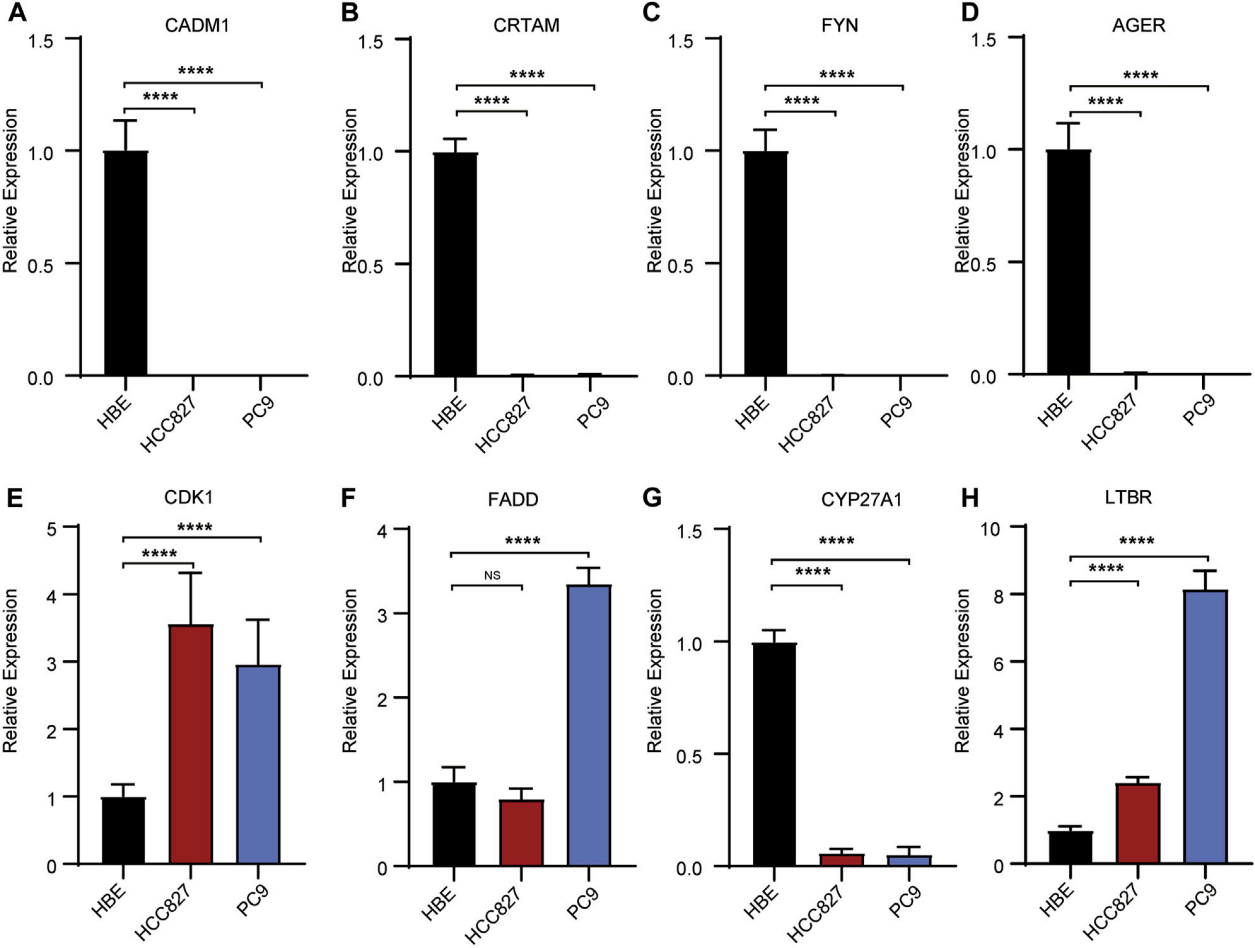
Validation of the expression of risk signatures in lung cancer cell lines (PC9 and HCC827) and normal lung cell (HBE) by RT-PCR analysis. **(A–D,G)** The expression of CADM1, CRTAM, FYN, AGER **(A–D)** and CYP27A1 **(H)** were decreased in normal lung cell lines. **(E, F, H)** The expression of CDK1, FADD **(E, F)** and LTBR **(H)** were unregulated in normal lung cell lines. *p < 0.05, **p <0.01, ***p < 0.001, ****p < 0.001.

To explore the clinical significance of the TPR signature, mRNA expression were validated in LUAD cell lines (PC9 and HCC827) and a normal lung cell line (HBE). As shown in Figure 13, the qPCR results indicated that the mRNA expression of *CDK1*, *FADD*, and *LBTR* were significantly increased in LUAD cell lines compared with those in the normal lung cell line, whereas the expression of *CADM*, *CRTAM*, *FYN*, *AGER*, and *CYP27A1* exhibited the opposite trend. No statistical difference was observed in the expression of *ADA* between LUAD cells and normal lung cells.

## Discussion

LUAD is characterized by multiple mutations and copy number alterations (Chen et al., 2020), posing challenges to establishing individualized immunotherapy. However, advances in immunotherapies that target the components of the TME have exhibited variable efficacy in the treatment of lung cancers, including LUAD (Kwak et al., 2018; O’Donnell et al., 2019). T cell functions are usually inhibited in cancers because of transcriptional and translational modifications introduced by other cell populations in the TME. The cytokines, chemokines, and nutrients in the TME enable cancer cells to escape from antitumor T cells (Speiser et al., 2016). Thus, secondary immunosuppression contributes to multiple biological processes involved in tumor progression and initiation. Recently, numerous positive TPRs have been identified *via* genome-scale screening, providing new insights into T cell therapy (Legut et al., 2022). TPRs are optimal targets for immunotherapy and may be closely related to the prognosis of LUAD patients.

In this study, nine TPRs in patients with LUAD were identified, using LASSO-Cox regression analysis. Most of these TPRs are differentially expressed in LUAD and are correlated with prognosis. For example, *FADD* overexpression affects NF-κB activity and cell cycle progression and is correlated with poor clinical outcomes in LUAD (Chen et al., 2005). *CADM1* is downregulated by miR-423-5p in LUAD tissues and cell lines, contributing to proliferation and metastasis (Huang and Feng, 2021). *CYP27A1* downregulation enhances the effect of cholesterol on LUAD cell proliferation and invasion and reduces high cholesterol-induced LUAD metastasis *in vivo* (Li et al., 2022). *FYN* expression in LUAD correlates with a poor prognosis and is downregulated in LUAD tissues and cell lines (Xue et al., 2020). *CDK1* upregulation correlates with poor prognosis, poor survival until first progression, and poor post-progression survival in patients with LUAD (Li et al., 2020). Downregulation of *AGER* (Zhang et al., 2018) and upregulation of *LTBR* (Zhang et al., 2019) are also correlated with the prognosis of LUAD, as demonstrated by multiple bioinformatics analyses. Our RT-PCR results were consistent with the documented downregulation of CRTAM. However, the role of ADA in LUAD has not yet been elucidated, and further studies are needed.

Three distinct TPC subgroups were identified by unsupervised consensus clustering. These three clusters exhibited different TME immune cell infiltration levels, biological pathway enrichment, and drug sensitivities. Cluster 1 was characterized by moderate T cell immune infiltration and activation of carcinogenic pathways, while cluster 2 was characterized by robust T cell immune filtration and the enrichment of pathways associated with carcinogenic gene sets and tumor immunity. Furthermore, cluster 2 also exhibited a robust positive correlation with immune activation-related biological processes, including cytokine-cytokine receptor interaction, interferon-gamma response, and inflammatory response. Additionally, cluster 2 had the highest expression scores for four immune checkpoint genes (PDCD1, PDCD1LG2, CTLA4, and LAG3) involved in immune blockage (Masugi et al., 2017; Solinas et al., 2019; Ali et al., 2020). The cluster 2 also exhibited moderate T cell exhaust and higher T cell activation score calculated by ssGSEA algorithm (Figures 4J,K). Therefore, this cluster may be the most suitable for immunotherapy. However, cluster 2 did not show a matching survival advantage (Figure 2). Although cluster 2 is a T-cell activated state (CD8^+^ T cell), the higher stromal cell activation indicates that high levels of CD4(+) T-cell-mediated, Treg infiltration and dendritic cell (poor antigen presentation capacity) may be induced immunosuppressive effect (Supplementary Figure S1) (Wang et al., 2012).

These results also indicate that cluster 2 can be classified as an immune-inflamed phenotype. Cluster 1 represents the intermediate phenotype, and the prognostic analysis results indicated that this cluster was associated with the best prognosis of the three. We attributed this result to the optimal localization and migration of T cells, which is essential for immune surveillance and the inhibition of tumor initiation (Smyth et al., 2016). In contrast, cluster 3 was negatively associated with the pathways linked to cluster 2 and featured low levels of immune cell infiltration, which is associated with immune tolerance and ignorance. Our analyses indicated that the dense stromal status in cluster 3 might influence the migration and activation of T cells, resulting in an immune-desert phenotype (Kim and Chen, 2016). To confirm the above findings, we performed a validation study in an independent TCGA-LUAD cohort. By analyzing TME immune cell infiltration and conducting enrichment analyses, we demonstrated the reliability of the TPCs for the identification and classification of immune phenotypes.

We have shown that TPRs are crucial mediators of multiple T cell functions and adaptive immune responses. However, we were unable to apply TPC analysis to individuals, as TPCs are a population-based tool. To account for the individual heterogeneity and complexity, a TPR risk model was established as a scoring system to evaluate and quantify the TPR patterns of individual LUAD patients. The low-risk group was classified as the enhanced T cell infiltration phenotype. This group was enriched in immune-related signaling pathways and was associated with a better prognosis. Conversely, the high-risk group was classified as the immune-excluded phenotype and was enriched in stromal cell-associated pathways, which restrict T cell entry into tumor islets by inhibiting their migration and penetration (Salmon et al., 2012). In addition, cluster 2, characterized by an immune-inflamed phenotype, was associated with a higher risk and a poor prognosis, whereas cluster 1 exhibited a lower risk and a better prognosis. These results demonstrated the feasibility and reliability of the risk model for assessing TPR patterns and prognosis in individuals with LUAD.

Checkpoint blockade therapy has shown surprising efficacy in the treatment of multiple cancers, especially in patients with an immune-inflamed TME (Kim and Chen, 2016; Cao et al., 2021). However, immune escape remains a major obstacle to achieving an extended OS in patients with solid tumors, including LUAD (Anichini et al., 2020). Many factors contribute to immune escape in LUAD, such as impaired antigen presentation, loss of heterozygosity in the human leukocyte antigen region, neoantigen silencing, and activation of immune checkpoints (Gajewski et al., 2013; Anichini et al., 2020). We successfully employed TPC analysis to distinguish among the immune phenotypes of the LUAD patients. We hypothesized that TPCs are associated with TMB and that TPCs can be used to predict the clinical response to checkpoint blockade immunotherapies. Consistent with previous reports, patients in cluster 2 with a high TMB (>10 Mb) and low TIDE score had a better response to PD-1/PD-L1 blockade therapy (Figures 4G–J) (Chan et al., 2019). In addition, the low-risk group was more susceptible to ipifarnib and nilotinib, both of which inhibit PD-1/PD-L1 directly or indirectly (Jackson et al., 1986; Tracy et al., 2022). Altogether, our results confirmed that TPCs are a valuable tool for predicting drug sensitivity and immunotherapy responses in patients with LUAD.

In summary, the TPR-related risk model exhibited reliability when used to evaluate the mutation features, degree of immune infiltration, and clinicopathological characteristics of individuals with LUAD. Moreover, the risk score served as a prognostic factor for predicting the prognosis of patients with LUAD and as a predictive factor for drug sensitivity. By developing a TPR-related risk model, our study provides novel insights into immunotherapy strategies.

## Data Availability

The datasets presented in this study can be found in online repositories. The names of the repository/repositories and accession number(s) can be found in the article/Supplementary Material.

## Supplementary material

SUPPLEMENTARY FIGURE S1 The immune infiltration of TPS subtype.
