# Supplementary figures and images for: Positive regulators of T cell proliferation as biomarkers for predicting prognosis and characterizing the immune landscape in lung adenocarcinoma
Source: Front Genet. 2022 Nov 25;13:1003754. doi: 10.3389/fgene.2022.1003754 (PMC9732442; doi:10.3389/fgene.2022.1003754)

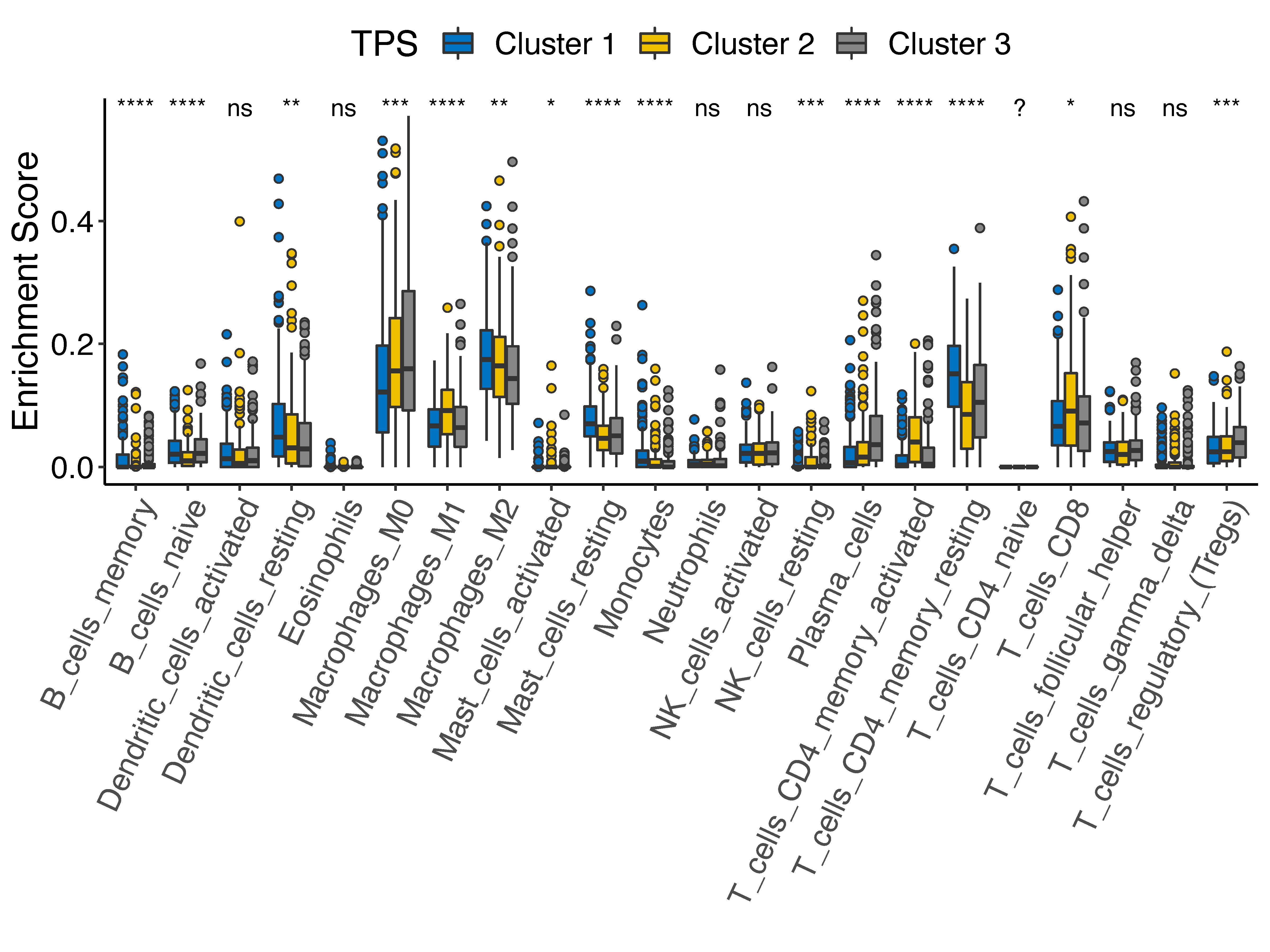

Supplement: Supplementary file 3 [file Image1.JPEG]
